# Genes Involved in Vasoconstriction and Vasodilation System Affect Salt-Sensitive Hypertension

**DOI:** 10.1371/journal.pone.0019620

**Published:** 2011-05-09

**Authors:** Lorena Citterio, Marco Simonini, Laura Zagato, Erika Salvi, Simona Delli Carpini, Chiara Lanzani, Elisabetta Messaggio, Nunzia Casamassima, Francesca Frau, Francesca D'Avila, Daniele Cusi, Cristina Barlassina, Paolo Manunta

**Affiliations:** 1 Division of Nephrology and Dialysis, San Raffaele Scientific Institute, Università “Vita-Salute” San Raffaele Hospital, Milan, Italy; 2 Department of Medicine, Surgery, and Dentistry, AO San Paolo, University of Milan, Milan, Italy; 3 Graduate School of Nephrology, University of Milan, Milan, Italy; 4 Genomics and Bioinformatics Unit, Fondazione Filarete, Milan, Italy; University of Cambridge, United Kingdom

## Abstract

The importance of excess salt intake in the pathogenesis of hypertension is widely recognized. Blood pressure is controlled primarily by salt and water balance because of the infinite gain property of the kidney to rapidly eliminate excess fluid and salt. Up to fifty percent of patients with essential hypertension are salt-sensitive, as manifested by a rise in blood pressure with salt loading. We conducted a two-stage genetic analysis in hypertensive patients very accurately phenotyped for their salt-sensitivity. All newly discovered never treated before, essential hypertensives underwent an acute salt load to monitor the simultaneous changes in blood pressure and renal sodium excretion. The first stage consisted in an association analysis of genotyping data derived from genome-wide array on 329 subjects. Principal Component Analysis demonstrated that this population was homogenous. Among the strongest results, we detected a cluster of SNPs located in the first introns of *PRKG1* gene (rs7897633, p = 2.34E-05) associated with variation in diastolic blood pressure after acute salt load. We further focused on two genetic loci, *SLC24A3* and *SLC8A1* (plasma membrane sodium/calcium exchange proteins, NCKX3 and NCX1, respectively) with a functional relationship with the previous gene and associated to variations in systolic blood pressure (the imputed rs3790261, p = 4.55E-06; and rs434082, p = 4.7E-03). In stage 2, we characterized 159 more patients for the SNPs in *PRKG1*, *SLC24A3* and *SLC8A1*. Combined analysis showed an epistatic interaction of SNPs in *SLC24A3* and *SLC8A1* on the pressure-natriuresis (p interaction = 1.55E-04, p model = 3.35E-05), supporting their pathophysiological link in cellular calcium homeostasis. In conclusions, these findings point to a clear association between body sodium-blood pressure relations and molecules modulating the contractile state of vascular cells through an increase in cytoplasmic calcium concentration.

## Introduction

Cardiovascular diseases are the major cause of health burdens and costs worldwide and essential hypertension accounts for about 50 percent of them [1]. Epidemiological, clinical and experimental studies support the relationship between dietary salt, renal salt handling and blood pressure (BP), suggesting dietary sodium (Na^+^) as an important contributor to hypertension [2]. However, BP response to changes in salt intake, namely salt-sensitivity, is very heterogeneous among individuals and is present in approximately half of the hypertensives [3]. Most importantly, salt-sensitivity is a negative prognostic indicator since it increases the risk for cardiovascular complications and their prognostic target organ damage indicators as left ventricular hypertrophy, microalbuminuria and endothelial dysfunction [4], [5]. Body Na^+^ and BP regulation is achieved through the interaction of several mechanisms, starting from Na^+^ handling in kidney tubular cells, moving to the myogenic tone at vascular level [6]. Further influences due to physical, nervous, and hormonal factors modulate this constitutive capacity of tubular cells to transport Na^+^ according to the body's needs [7].

Salt-sensitive hypertension is, at least in part, under genetic control [8] but the underlying genetic mechanisms are not fully clarified. Salt-sensitivity can be assessed with different standardized protocols including acute salt load, chronic dietary sodium depletion and chronic sodium volume depletion. Our group performed a systematic analysis of the genetic mechanisms involved in salt-sensitivity, using a well-standardized protocol, consisting in the acute intravenous infusion of saline. So far we have evidenced the genetic contribution of *ADD1*, *ACE*, *WNK1* and *NEDD4L* variants to the acute BP response [9], [10], [11], [12].

For the present, and the next steps, we decided to exploit a technique alternative to the manual TaqMan, used previously, i.e. that of low density genome-wide genotyping array. This has been done not necessarily to have a complete view of all the genome, but for the following two reasons: (i) to get rid of the uncertainty of potential hidden stratification that could affect the results and correct for it, if needed; (ii) to have our data set genotyped once, and to concentrate on a specific hypothesis that involves one genetic mechanism or pathway at a time [13].

We are well aware that Genome-Wide Association studies (GWAs) represent a powerful tool to explore variants associated to complex traits and to evaluate possible gene-gene interactions [14], but the huge number of samples needed to reach the genome-wide threshold of significance cannot be realistically applied to an endophenotype like salt-sensitivity, which requires great care in measurement. In fact, among the studies published so far on the genetics of essential hypertension [15], [16], [17], [18], [19], [20], no GWA data are available on the genetics of salt-sensitive hypertension.

Here, we report an association analysis with a genome-wide genotyping array (stage 1) in a selected population of limited sample size but consisting on mild hypertensive Caucasians who underwent an acute salt load with the aim to search for genes related to BP changes after this manoeuvre. Large-scale genotyping enabled a systematic analysis of genetic susceptibility to salt-sensitive hypertension and possible imputation. The genome-wide genotyping data were also used to perform a Principal Component Analysis (PCA) to assess the genetic variance between the samples. After genomic control, PCA analysis demonstrated that this population was homogeneous. Among the strongest results we identified genes that code for proteins involved in the vasoconstriction/dilation system at different levels. The relevant SNPs were replicated in an additional cohort (stage 2) and in the combined analysis. The genes identified, *PRKG1*, *SLC24A3* and *SLC8A1*, influence the vascular tone likely through an increase in the concentration of cytoplasmic calcium (Ca^2+^), and may represent a significant key node for systemic BP regulation.

## Results

We performed an association analysis with a genome-wide genotyping array (stage 1) for three quantitative traits (ΔDBP120, ΔSBP120 and PNat120; see Methods for details) measured in a subset of hypertensive patients (n = 329) who underwent an acute salt load. The three quantitative traits showed a normal distribution. Subsequently, in stage 2, as replication, we genotyped only the SNPs found significantly associated in stage 1 in 159 other hypertensives who underwent the same test. These data were then evaluated in a combined analysis. The clinical characteristics of all subjects are shown in Table 1.

**Table 1:** Clinical characteristics of 474 patients underwent an acute test of salt loading.

	Mean±s.d.
Sex (m/f)	401/73
Age (years)	51.4±10.6
BMI (kg/m^2^)	25.9±3
24 h SBP day-time (mmHg)	143.2±12.3
24 h DBP day time (mmHg)	93.8±9.5
24 h Ur. Na^+^ (mEq/L)	144.2±69
24 h Ur. K^+^ (mEq/L)	58.7±23.3
ΔSBP120	4.3±9.7
ΔDBP120	2±6.5

Abbreviations: BMI, body mass index; Ur. Na^+^ and Ur. K^+^, urinary Na^+^ and K^+^ excretion; 24 h refers to 24 hour Ambulatory BP monitoring measurements.

### Association analyses in stage 1

**Figure 1:**
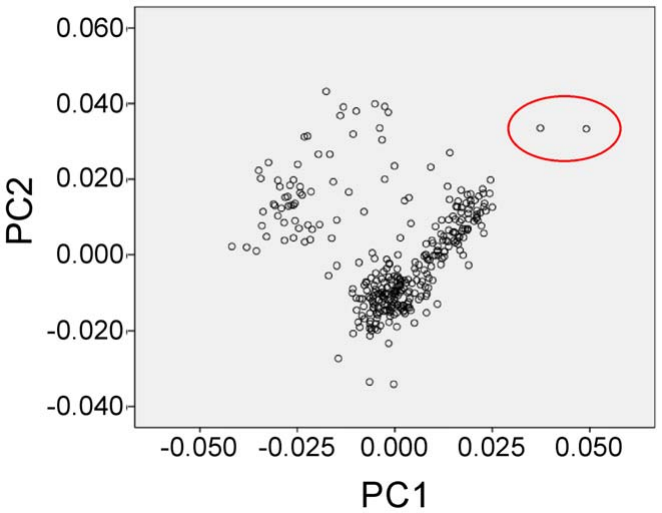
Multidimensional scaling plot of stage 1 cohort. Individuals in red circle were identified as outliers and subsequently excluded. The two principal components are plotted on the axis.

Genome-wide genotyping array allowed to screen a huge number of genetic nucleotide variations simultaneously and to perform a genomic control of the first set of 329 subjects (stage 1). PCA method is applied to large-scale association studies based on SNP both to quantify population relationships and to correct for population stratification [21], [22]. PCA identified two outlier individuals that were subsequently excluded from the study, thus indicating that population stratification was negligible (Figure 1). In addition, the test of population homogeneity confirmed that our population was homogeneous, as the clusterization of individuals was not significant (lambda = 1.0032 for ΔDBP120, lambda  = 1.0044 for ΔSBP120 and lambda = 1.0008 for PNat120). PCA was also performed including Tuscans and Utah residents with Northern and Western European ancestry population of HapMap data and our cohort resulted in a perfect overlap with the Italian population (Figure S1).

After quality control (Table S1), we analyzed 293,195 SNPs (MAF≥0.05) in 319 subjects. First, we tested all SNPs using an unbiased association design. Mainly considering the limited sample size and the practical impossibility to increase its size, we used an adaptive permutation procedure to control the type I error [23].

Among the top ranking association signals we first annotated SNPs mapping in well-characterized molecules affecting BP or in genes previously found associated with BP. After whole analysis, we focused on SNPs and genes involved in the control of cytoplasmic Ca^2+^ concentration, being the control of vascular tone a key node for systemic BP regulation. Table 2 reports the association of SNPs in *PRKG1* and *SLC24A3* genes associated with ΔDBP120, ΔSBP120 and PNat120.

**Table 2:** Association of top ranking SNPs in *PRKG1* and *SLC24A3* with ΔDBP120, ΔSBP120, PNat120 in stage 1 (n = 319).

	*PRKG1*			*SLC24A3*
Top SNP	rs1904694	rs7897633	rs7905063	rs6112470
position (chr)	52905494 (10)	52957721 (10)	52964590 (10)	19562579 (20)
risk allele (frequency)	G (0.42)	A (0.52)	T (0.53)	C (0.15)

*Regression coefficient (p value)*				
ΔDBP120	1.95±0.49 (8.17e-05)	2.06±0.48 (2.34e-05)	1.87±0.47 (9.67e-05)	ns
ΔSBP120	ns	1.68±0.77 (0.029)	1.62±0.76 (0.033)	4.75±1.15 (4.28e-05)
PNat120	ns	ns	ns	0.015±0.004 (6.39e-04)

Data generated by Plink assuming an additive genetic model, with a p-value smaller than E-04 for at least one of the phenotypes analyzed. Permutation was performed and maintained the same robustness of significance.

Effect sizes are on the mmHg scale (±s.e.) (except for PNat120, µEq/mmHg/min) for increasing copy of the risk allele as estimated by the beta coefficient in linear regression.

Position on chromosome refers to 37.1 Genome Build of GRCh37 (http://www.ncbi.nlm.nih.gov/SNP).

#### PRKG1

Three SNPs in *PRKG1* gene (rs1904694, rs7897633 and rs7905063), spanning 59 kb from intron 1 to intron 2 at 10q11.23, showed a striking cluster of association with ΔDBP120 (p value = 8.17E-05, 2.34E-05 and 9.67E-05 under additive genetic model, respectively) (Figure S2). The effect size on DBP variation (beta coefficient) for each copy of susceptibility allele for salt-sensitivity ranged from 1.87 to 2.06 mmHg (Table 2). *PRKG1* codes for type I cGMP-dependent protein kinase, a nitrovasodilator effector that has been shown to mediate vascular smooth muscle cells (VSMC) relaxation by lowering intracellular Ca^2+^level [24], [25].

LD block and tagging analysis (r^2^≥0.9) of the three *PRKG1* top SNPs in our samples indicated that they map in different haplotype blocks (Figure S3). Particularly, rs7897633 SNP did not show any proxy and the other two SNPs did not result in LD with known informative polymorphisms, as well, according to CEU HapMap Phase II reference panel. We next used imputation to expand the set of investigated SNPs and search for possible functional variants. None of the 655 imputed SNPs gave better signals of association.

Comparative analysis revealed a high conservation of the *A* allele of rs7897633 within a moderately conserved flanking intronic region, denoting the ancestral nature of this nucleotide (Figure S4). Interestingly, the *A* allele is the risk allele for salt-sensitive response to Na^+^ intake (ΔDBP120 in *AA* +4.73±0.71, mean±se), whereas the protective *C* allele did not significantly influence BP response to salt load (ΔDBP120 in *CC* +0.67±0.67, mean±se) and appears only in humans. Moreover, in HapMap populations the *C* allele is present at high frequency (∼0.50) both in Europeans and in Asians, and at very low frequency in Africans (http://www.ncbi.nlm.nih.gov/SNP/snp_ref.cgi?rs=rs7897633).

#### SLC24A3

A subsequent analysis also revealed a top ranking SNP, rs6112470, in intron 4 of *SLC24A3* gene and associated to both ΔSBP120 (p = 4.28E-05; effect size = 4.75±1.15 mmHg) and PNat120 (p = 6.39E-04; effect size = 0.015±0.004 µEq/mmHg/min) under additive genetic model, with a salt-sensitive effect of minor *C* allele (Table 2). *SLC24A3* codes for the NCKX3, the potassium dependent Na^+^/K^+^/Ca^2+^ exchanger type 3, an important regulator of intracellular calcium homeostasis [26]. Pairwise correlation in HapMap CEU (r^2^>0.9) of rs6112470 included other six SNPs in nearby intron 3 and 4. When we enlarged the analysis to the complete gene region, a cluster of association of SNPs spanning 526 kb starting from the end of intron 2 till mid intron 5 was identified even if with a p value range (7.8E-03_1.1E-04) (Figure S5). Imputing revealed that among the 394 SNPs examined across this region, the imputed SNP rs3790261 had the strongest association signal with ΔSBP120 (p = 4.55E-06), compared to any of the initial top ranking SNPs. Pairwise correlation between the original and the imputed SNP was 0.832 (r^2^) calculated on our dataset. As rs3790261 is a synonymous change located at position +20 of exon 4 (*c.369G>A*), we performed an *in silico* analysis with ESEfinder, and we observed that the minor *G* allele abolished an exonic splicing enhancer (ESE) for SRp40 binding site (Figure S6). We then considered rs3790261 for further analysis.

#### SLC8A1

SLC8A1, that codes for Na^+^/Ca^2+^ exchanger type 1 NCX1 is one of the best candidate molecule in the biochemical control of peripheral vascular resistance and its role in the pathogenesis of hypertension and salt-sensitivity has been previously demonstrated [27]. It belongs to plasma membrane Na^+^/Ca^2+^ exchange proteins, with similar physiologic characteristics to NCKX3, except for K^+^ dependence [28]. Therefore, it was obvious to look at our association results for *SLC8A1* in order to extend previous findings. An interesting cluster of association with more than one quantitative trait was identified. The statistically significant association between rs434082 and ΔSBP120 was specific for this trait (p = 4.7E-03), while rs11893826 was also associated to PNat120 (p = 0.018) and had the weaker r^2^ in relation to rs434082 (Table 3). Particularly, all these SNPs are located within the long intron 1 covering a region of about 105 kb, and LD pattern reported in Figure S7 refers to canonical matrix with high recombination rate. For each significant SNP a functional proxy was searched with imputation, without improving the results.

**Table 3:** Candidate SNPs in *SLC8A1* significantly associated with DSBP120 (A), and LD r2 pattern between the best associated SNP (rs434082) and the other SNPs (B).

A		
	rs434082	rs11893826
position	40485074	40564647
risk allele (frequency)	A (0.08)	A (0.40)

*Regression coefficient (p value)*		
ΔSBP120	3.5±1.23 (0.0047)	2.15±0.86 (0.012)
PNat120	ns	0.008±0.003 (0.018)

B	
SNP	r^2^# rs434082
rs666362	0.702
rs487119	0.229
rs403630	0.133
rs10490053	0.067
rs727477	0.066
rs6544318	0.056
rs4952404	0.042
rs11893826	0.006

(A) Data generated by Plink assuming an additive genetic model. Permutation was performed and maintained the same robustness of significance. Effect sizes are on the mmHg scale (±s.e.) (except for PNat120, µEq/mmHg/minute) for increasing copy of the risk allele as estimated by the beta coefficient in linear regression.

Position on chromosome 2 refers to 37.1 Genome Build of GRCh37 (http://www.ncbi.nlm.nih.gov/SNP).

(B) #Linkage Disequilibrium between rs434082 and other SNP associated with ΔSBP120 trait.

### Stage 2 Association and Combined analyses

In stage 2 we performed genotyping in an additional population of 159 subjects, for SNPs in *PRKG1* (rs1904694, rs7897633, rs7905063), *SLC24A3* (rs3790261) and in *SLC8A1* (rs11893826, rs434082). As shown in Table S2, we did not obtain any significant association except for *PRKG1* locus, reflecting the limited power of such a small cohort, though the trend of the allelic effect was maintained across the two-stage studies.

Being the selection criteria of the patients the same between stage 1 and 2, we performed a combined analysis. As we observed some differences for sex, age, BMI between patients of the two stages, results were adjusted for these variables. Regression analysis on the combined data revealed an effect size on SBP variation (beta coefficient) for each copy of susceptibility allele for salt-sensitivity of 3.67 mmHg for rs3790261 (*G* compared to *A*) in *SLC24A3* (p = 2.97E-05), 1.87 mmHg, and 3.16 mmHg SBP for *SLC8A1* rs11893826 (*A* compared to *G* - p = 5.81E-03) and rs434082 (*A* compared to *G* - p = 8.51E-04), respectively. For *PRKG1* SNPs the effect size was 1.36 mmHg on DBP change for rs1904694 (*G* compared to *A* - p = 1.50E-03), whereas rs7897633 (*A* compared to *C*) and rs7905063 (*T* compared to *C*) exhibited similar effect sizes of 1.86 and 1.84 mmHg (p = 1.28E-05 and 1.90E-5) respectively (Table 4). For these last SNPs the risk allele was the most frequent.

**Table 4:** Association of SNPs in *PRKG1*, *SLC24A3* and *SLC8A1* with ΔDBP120, ΔSBP120 in combined analysis (n = 478).

Gene	SNP	risk allele	beta (se)	p value	phenotype
*PRKG1*	rs1904694	G	1.36 (±0.43)	1.50E-03	ΔDBP120
	rs7897633	A	1.86 (±0.42)	1.28E-05	ΔDBP120
	rs7905063	T	1.84 (±0.43)	1.90E-05	ΔDBP120
*SLC24A3*	rs3790261	G	3.67 (±0.87)	2.97E-05	ΔSBP120
*SLC8A1*	rs11893826	A	1.87 (±0.67)	5.81E-03	ΔSBP120
	rs434082	A	3.16 (±0.94)	8.51E-04	ΔSBP120

Effect sizes are on the mmHg scale (±s.e.) for increasing copy of the risk allele as estimated by the beta coefficient in linear regression based on the additive genetic model.

Data adjusted for sex, age, BMI and stage.

The likely increased activity of both Ca^2+^ cotransporters due to the susceptibility alleles for salt-sensitivity has been evaluated in all population. No difference was observed if we examined total plasma Ca^2+^. We then analyzed the effect of *SLC8A1* rs11893826 and *SLC24A3* rs3790261 variants in relation to urinary Ca^2+^ excretion (expressed as UCa^2+^/urinary creatinine*plasma creatinine, mg/100 ml of glomerular filtration rate, GFR): the former resulted significantly associated at t 120 after Na^+^ load (non risk *GG* 4.75±0.31 vs risk *GA+AA* 3.70±0.16 mg/100 ml GFR, p = 0.001), indicating a reduced Ca^2+^ excretion for susceptibility variant. Similar results were observed for *SLC24A3* rs3790261 both at t 0 (non risk *AA* 2.25±0.18 vs risk *AG+GG* 1.58±0.16 mg/100 mL GFR, p = 0.021), and t 120 (non risk *AA* 4.35±0.21 vs risk *AG+GG* 3.67±0.23 mg/100 ml GFR, p = 0.045).

### Interaction analyses

The vascular tone is determined by a certain number of proteins that are part of a common pathway regulating force generation by the actin-myosin-based contractile apparatus of the cell. Abnormalities of this system can cause primary changes in VSMC tone and affect BP. Therefore, we searched for potential interactions (i.e., deviation from an additive model) in the complete data set among the genes identified as significantly associated to BP changes after salt load in single SNP association.

**Figure 2:**
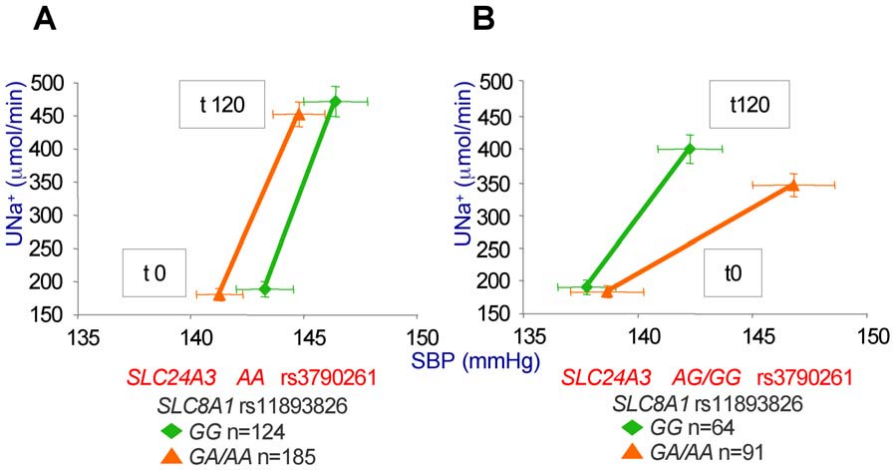
Renal pressure-natriuresis relationship during acute salt load according to the rs3790261 (*SLC24A3*), rs11893826 (*SLC8A1*) genotypes. Urinary sodium excretion (U_Na+_) as a function of SBP, at basal level (t 0) and after salt load (t 120). The pressure-natriuresis curve is drawn in the left panel considering *SLC8A1*, rs11893826 polymorphism (carriers of the non risk allele *G*, green slope, and carriers of the risk allele *A*, orange slope), in the genetic background of the non risk *SLC24A3 AA* genotype at rs3790261. It is drawn in the right panel considering the same genotypes for *SLC8A1*, but within the genetic context of the risk *SLC24A3 G* allele at rs3790261. The significantly less steep slope of the orange line in the right panel, compared to the three other slopes indicates the need for BP increase in order to excrete the salt load in carriers of that specific allele combination.

In 2-SNP analyses, assuming a dominant genetic model, we observed a significant interaction between rs3790261 in *SLC24A3* and rs11893826 in *SLC8A1* affecting absolute changes in SBP and Na^+^ excretion in response to 120 minutes of salt loading (PNat120) (p interaction = 1E-03, p model = 9.12E-05) (Figure 2). The slope of the pressure-natriuresis relationship was less steep in patients who carried both minor alleles (*AG/GG* for rs3790261 and *GA/AA* for rs11893826; 0.031±0.005 µEq/mmHg/min ± se) than in those carrying the remaining genotype combinations (0.010±0.002 µEq/mmHg/min ± se) (p interaction = 1.55E-04, p model = 3.35E-05 in dummy analysis). The significantly different slopes of the two curves indicate that a larger increase of SBP was needed in carriers of both minor (risk) alleles to excrete the same amount of Na^+^, i.e. such subjects are salt-sensitive.

## Discussion

We conducted an association analysis on BP response to an acute salt infusion in a subset of accurately phenotyped hypertensive patients. This manoeuvre allows to monitor the simultaneous changes in BP and renal Na^+^ excretion to evaluate their relationship and to define the individual salt-sensitive condition. Two different methods are mainly used to diagnose salt-sensitivity: acute intravenous saline load and changes in dietary salt intake, generally from high NaCl intake (300 mEq/die) to low Na (<40 mEq/day) for 7 to 14 days [29]. In the latter method a low compliance is more frequent than in acute sodium load because strict dietary manipulations are difficult to achieve. This is a relevant point because it can affect the accuracy of diagnosis.

The present study is a two-stage analysis: we used a genome-wide genotyping array in the first set of subjects (stage 1) primarily to verify the homogeneity of our population by several statistical analysis like PCA, homogeneity test, and to perform imputation as a huge number of nucleotide variations were screened simultaneously. Stage 2 can be considered a replication of the first one, though all patients were enrolled in our clinical unit.

In stage 1 we obtained some appealing results concerning variants in genes involved in vasoconstrictor/dilator processes. The genes we focused on, *PRKG1*, *SLC24A3* and *SLC8A1*, regulate intracellular Ca^2+^ concentration and through this mechanism can control vascular tone that is a key node for systemic BP regulation [6], [7]. First, we found a strong association with variation in DBP after acute salt load for a cluster of SNPs mapping in the first introns of *PRKG1* gene. This association was then confirmed in the combined analysis (rs7897633, p = 1.28E-05). Second, a detailed annotation of top ranking SNPs identified *SLC24A3* and *SLC8A1* associated to variation in SBP (the imputed rs3790261, p = 2.97E-05; and rs434082, p = 8.51E-04). Both genes code for plasma membrane Na^+^/Ca^2+^ exchange proteins (NCKX3 and NCX1, respectively) with similar physiologic characteristics, except for K^+^ dependence. This physiological link was also supported by a significant epistatic genetic interaction between the two loci on PNat120 in the combined analyses. Only carriers of the risk alleles, the minor alleles in both cases, showed a salt-sensitive pressure-natriuresis curve (p interaction = 1.55E-04, p model = 3.35E-05).

These results suggest that BP modifications acutely induced by the salt load involve genes regulating contractility of the VSMC either through an increase in intracellular free Ca^2+^ concentration or through an increased sensitivity to Ca^2+^ of the contractile elements in response to various signalling events. Indeed, Ca^2+^ plays a central role in controlling vascular tone, thereby making significant contributions to the regulation of systemic BP [30], [31].

The most interesting SNP of our study, both in terms of association and possible evolutionary impact, is rs7897633 at *PRKG1* locus. In fact, the *A* allele, the risk allele for salt-sensitivity, reflects the ancestral status, whereas the protective *C* allele is present exclusively in humans and is more frequent in non-African populations. Other variants in genes involved in the control of sodium homeostasis (*AGT* and *CYP3A5* genes) show a similar pattern: ancestral alleles are linked with an increased risk to hypertension and are fixed in several primates, while the derived protective alleles appear to carry the signature of positive natural selection with a quick raise to high frequency [32].

*PRKG1* encodes for the type 1 cGMP-dependent protein kinase (cGKI), a nitrovasodilator effector widely distributed in eukaryotes. Examining gene expression pattern in cultured VSMCs, Tamura et al [33] detected an abundant human alpha subtype mRNA in the aorta, heart, kidney and adrenals. The alpha and beta isoforms that differ in their amino termini, exert their simultaneous activity as mediators of smooth muscle tone relaxation in vascular and nonvascular smooth muscle. cGKIs are modulators of cell growth as well. Changes in vascular morphology and tone can increase vascular resistance and BP [34], and may alter cardiac contractility and remodelling. A recent study indicates that *PRKG1* impinges on circadian rhythms, influencing a multitude of physiological processes such as cardiovascular activity [35]. Another study in a Chinese population supported the hypothesis that the abnormalities of VSMCs that alter the intrinsic contractile state of the cell can directly cause abnormal vascular tone and disorders of BP regulation [36]. In this study a four-gene interaction model of association that includes *PRKG1* was found to affect the risk for essential hypertension [36]. A conventional deletion of the gene for cGKI has been studied in mice and multiple phenotypes, including elevated BP, have been observed [37]. Mice with a selective mutation in the N-terminal protein interaction domain of cGKI-alpha display inherited vascular smooth muscle cell abnormalities of contraction, abnormal relaxation of large and resistance blood vessels, and increased systemic BP [34]. These mice express mutant cGKI-alpha protein that is incapable of binding to myosin light chain phosphatase, one key protein of vascular smooth muscle cell (VSMC) contractile apparatus. From these data we expected that *PRKG1* gene does not directly affect renal sodium transport, despite we observed a significant association with the difference in DBP after acute Na load. Particularly, the risk alleles may influence the vascular tone likely through an abnormal elevation in vascular smooth muscle tone, leading to smooth muscle dysfunction.

The intronic SNP rs6112470 in *SLC24A3* is associated to SBP variation in our study. However, from the functional point of view, the most informative SNP was the imputed rs3790261 in exon 4, since it resulted in an effect size on BP of 3.67 (p = 2.97E-05). Due to its synonymous nature it may be included in a specific short oligonucleotide sequence that affects exon splicing. ESEs within exons promote splicing of the corresponding exons and subsequent exclusion mediated by splicing regulatory proteins [38], [39]. An *in silico* search for inclusion of this polymorphism in a putative ESE for SRp40 binding site suggests that rs3790261 may affect the posttranscripional regulation of *SLC24A3* mRNA, thus representing a RNA splicing signal. Further experimental settings are needed to validate this hypothesis.

*SLC24A3* encodes for NCKX3, one of the six isoforms of Na^+^/K^+^/Ca^2+^ exchanger family, and can operate either in forward and in reverse mode, depending on both ion gradients and the membrane potential. Consequently, it represents one of the major contributors in regulating Ca^2+^ flux in environments that experience wide and frequent fluctuations in Na^+^ concentration [26], [28]. A recent gene expression microarray analysis in mice demonstrated that NCKX3 is expressed in the kidney, where it is primarily localized in the basolateral layer of the distal convoluted tubules, with no detectable expression in the glomerulus and proximal convoluted tubules, thus participating in active calcium transport in the kidney [40]. NCKX3 was previously described in various mammalian tissues, such as almost all regions of the brain, in aorta, uterus, and skeletal muscle [41].

*SLC8A1* gene may be phenotypically correlated with *SLC24A3* because it codes for NCX1 transporter. Its role in the pathogenesis of hypertension and sodium-sensitivity [27] has been already demonstrated and makes it one of the best candidate molecule in the biochemical control of peripheral vascular resistance. Indeed, we found a significant association with SBP variation for a cluster of intronic SNPs. NCX1 is abundantly expressed in kidney, heart, brain and smooth cells. In particular, mice with genetically engineered NCX1 proved that it contributed to long-term BP regulation resulting in increased BP when it is over expressed, or alternatively reduced BP in knockout [42]. Accordingly, a significant association with SBP and hypertension regarded *SLC8A1* locus in a recent study of candidate genes for BP regulation in KoraS3 cohort [13].

Our association results can also be considered as true positive on biological grounds. In fact, a common biological pathway for NCX1 and NCKX3 is shown as both genes are associated with the same phenotypic trait (variation in SBP after salt load) and display an epistatic interaction that affects PNat120. This is important since polygenic multifactorial diseases are likely caused by many alleles that confer a small individual effect but a high risk in combination. Therefore, a combined analysis with different SNPs exploring possible interactions can add interesting information. The variations in intracellular Ca^2+^ are involved in the vascular and tubular response to salt load [27]. Therefore, our results showing the influence of the polymorphisms of genes encoding Ca^2+^ transport system are consistent with these pathophysiological data. In this study we did not address Ca^2+^ renal excretion issues, but a preliminary analysis showed no association of *SLC8A1* and *SLC24A3* with the total plasma Ca^2+^. However, if urinary Ca^2+^ excretion was examined, a significant association was obtained for both exchanger variants (rs3790261 and rs11893826) at the end of infusion and at basal time only for *SLC24A3*, clearly indicating a reduced Ca^2+^ excretion caused by a likely gain of function of both risk variants of these cotransporters. To convey this important issue further study will be required on Ca^2+^ renal handling.

In the present study careful attention was paid to selection and phenotyping of the hypertensive patients and the same criteria were applied in stage 1 and stage 2 of the study. Only newly discovered and consequently never treated hypertensive patients were enrolled. These stringent selection criteria allowed excluding the confounding effects of antihypertensive therapy and hypertension duration. In most established association and GWA studies published so far, BP values reported are biased by the antihypertensive treatment ignoring the therapy or solving by adding 10–15 mmHg to the observed BP values, because the need of very large sample sizes doesn't always allow detailed enrolment [19], [20], [43]. The duration of hypertensive status is also important since the pathophysiological mechanisms of hypertension or organ damage are phase dependent [44]. Moreover, we performed a genomic analysis on whole genome data and demonstrated that our cohort was homogeneous and perfectly overlapping with another Italian cohort.

A limitation of our study is the small sample size that reflects on the weak association signals. This can certainly impact the results of the genetic interactions that are also dependent on large data-sets [45]. However, this limitation could be in part overcome by the use of a well characterized intermediate phenotype measurable as quantitative trait, that can add a considerable power compared to typical case-control approaches [46]. In fact, an intermediate phenotype (or endophenotype) of essential hypertension can help to reduce the number of genetic and environmental confounding interferences and increases the chance to identify the genetic variants that influence the phenotype [47].

In summary, our study reports an association analysis of genotyping data derived from genome-wide array on the BP response to salt load. We identified a cluster of variations associated with changes in both diastolic (rs1904694, rs7897633 and rs7905063) and systolic BP (rs3790261, rs434082 and rs11893826) consequent to salt load. The associated SNPs map in *PRKG1*, *SLC24A3* and *SLC8A1* that are involved in the vasocostriction/dilation system. The understanding of the molecular pathways involved in BP response to acute salt load provides new knowledge for possible mechanisms leading to cardiac and cerebral organ damage. Furthermore, these findings can help to classify hypertension into sub-phenotypes based on genotypic data for a gene-driven personalized therapy.

## Materials and Methods

### Overall study design

This study was arranged in two stages: 329 patients underwent the acute salt loading test and were included in the discovery stage (stage 1). Subsequently, further 159 patients underwent the same salt loading test and were genotyped to confirm specific initial findings (stage 2). Data from the two cohorts were combined to provide a robust estimate of effect size for SNPs associated in stage 1. The San Raffaele Ethics Committee approved the study and all participants gave the written informed consent.

All patients were newly discovered, never treated hypertensives enrolled in the ‘Outpatient Clinic for Hypertension’ of San Raffaele Hospital of Milan. In our selection criteria [48] we excluded women taking estroprogestinic pill, resulting in a low percent of women included in the study. Moreover, secondary forms of hypertension were ruled out by routine examinations. All individuals underwent an acute test of salt loading following the enrolment criteria of mean day-time SBP≥135 or DBP≥85 mmHg at 24 hours ambulatory blood pressure monitoring, age 18–65 years, and BMI<30 kg/m^2^. During a run-in period of 3–5 weeks, the patients were instructed to keep a constant Na^+^ intake of 150 mEq/24 h (which is the average Na^+^ intake observed in Milano [48]). Each patient collected 24 h urine for analysis of compliance to the diet on the day before hospital admission. Clinic basal BP as in-patient was recorded in the same morning when blood samples for plasma renin activity and aldosterone were drawn (on the day before the salt load). The study protocol for acute salt load test was similar to that previously reported [48].

The slope of the relationship between SBP and Na^+^ excretion (PNat pressure-natriuresis relationship, µEq/mmHg per minute) was calculated by plotting the Na^+^ excretion on the *y* axis as a function of SBP on the *x* axis measured both under basal conditions (time 0 = t 0) and after 2 hours of salt infusion (time 120 = t 120), as described elsewhere [10].

### Phenotype and Genotyping

For the analysis of association two main groups of phenotypes were used: 1) BP phenotype – ΔDBP120 or ΔSBP120 is the difference between Diastolic/Systolic BP at the end of salt load (at time 120) and at baseline (at time 0); 2) Pressure-natriuresis phenotype – PNat120 is the ratio between the ΔSBP120 and total Na^+^ excretion amount from baseline to 120. All phenotypes were analyzed as quantitative trait.

Genomic DNA was extracted from peripheral blood with standard method. Patients from stage 1 were genotyped with the Illumina (San Diego, CA) Infinium II HumanHap300 Duo BeadChips. The 318,237 tagSNPs on this chip are taken from Phase II of International HapMap Project and provide 80% coverage of common variation in European population (r^2^ = 0.80). The 9,035 SNPs on chromosome X are not included in this analysis. In total, 309,202 autosomal SNPs were genotyped.

Mean call rate per individual was 0.983; 2 of 329 individuals were removed for low genotyping (call rate<0.1). 5,437 SNPs with genotyping rate >0.1 (% SNPs not called) and 10,031 SNPs with MAF<0.05 were not included in the analysis. 1,876 markers failed Hardy-Weinberg (HW) test (p≤0.001). However, being our sample composed only by cases we did not apply any HW filter because these SNPs could be potentially causal. After frequency pruning and quality control the analysis was done on 293,195 SNPs for 319 samples (Table S1).

A further genotyping quality control was obtained by comparing genotypes obtained from the genotyping array mentioned above to those previously generated for the stage 1 samples in other studies (*NEDD4L* rs4149601 [10]; *ADRB2* rs1042713 and *AGT* rs699, not published). All these genotypes resulted in total agreement with the previous data obtained with other technique.

Genotyping in stage 2 was performed with the 5′ nuclease allelic discrimination assays, with allele-specific MGB probes (TaqMan SNP Genotyping Assay-Applied Biosystems, Foster City, CA, USA).

Missing genotypes were imputed with MACH version 1.0 (Markov Chain based haplotyper) [49]. Reference genome sequence for the imputation was downloaded from the International HapMap Project web site (HapMart) for both the CEU (Utah residents with Northern and Western European ancestry from the CEPH collection), and TSI (Tuscans in Italy) population.

### Statistical analysis

Univariate linear regression analyses were carried out using an additive genetic model of each filtered SNP using the statistical with package PLINK version 1.06 [50]. This kind of analysis is performed on the genotypic frequencies using different types of statistical tests. Furthermore, to avoid false positive findings (type I error) we requested adaptive permutations based on the Wald statistics [50]. Population stratification assessment was performed using PCA of eigenstrat and MDS as implemented in PLINK.

Association analysis in stage 2 was tested using one-way ANOVA under an additive genetic model. In combined analysis, we used General linear model (GLM) univariate analysis with continuous phenotypes as dependent variable, semi-continuous SNP genotype as independent variable under an additive model and study stage, sex, age and BMI as covariates for adjustment.

Each associated SNP was fitted in regression analysis as a linear term: the effect (beta) on BP in mmHg and on PNat in µEq/mmHg/min of the risk allele for Na^+^ sensitivity was assumed to be additive. We explored with GLM analysis if the effect of salt load on dependent variable (PNat120) depends on different genetic factors (independent variables; gene-gene interaction). In this case, the effect size was obtained by linear regression analysis using dummy variables based on the presence or absence of specific combination. SPSS (version 17 for MacOS X; SPSS Inc. Chicago, Illinois, USA) software was used for general statistical analysis.

The regional plot was generated using LocusZoom software (http://csg.sph.umich.edu/locuszoom/). Linkage pattern was evaluated by using Haploview 4.2 software (http://www.broad.mit.edu/mpg/haploview). Particularly, LD bins were estimated by Tagger algorithm implemented in Haploview.

*In silico* assessment of rs3790261 sequence for putative *cis*-acting ESE and ESS motifs was performed with he ESE Finder 3.0 [51] (http://rulai.cshl.edu/cgi-bin/tools/ESE3/esefinder.cgi?process=home).

## Supporting Information

Figure S1 Multidimensional scaling plot of stage 1 cohort (blue circles), Utah residents with Northern and Western European ancestry population from the CEPH collection (green circles) and Tuscans (red circles) of HapMap data Release 27. The two principal components are plotted on the axis.
(PDF)

Figure S2 Regional plot of the *PRKG1* and flanking region (chromosome 10 q11.23). The *P* values [−log(*P*)] of all genotyped SNPs annotated with the gene structure are indicated. The best top SNP for ΔDBP120 is marked with purple diamond and the rs code is also reported. The graph was drawn with LocusZoom software.
(PDF)

Figure S3 Map of the initial part of *PRKG1* gene. The three independent SNPs are in the red box. The LD value (r^2^) between a specific pair of SNPs is shown within a corresponding “square”. The graph was drawn with Haploview software (http://www.broad.mit.edu/mpg/haploview).
(PDF)

Figure S4 Multiple sequence alignment of rs7897633 region in intron 2 of *PRKG1*. The highlighted area indicates SNP position. M means *A/C* transversion. Sequence alignment is provided by the UCSC Genome Browser (http://genome.ucsc.edu).
(PDF)

Figure S5 Regional plot of the *SLC24A3* and flanking region (chromosome 20 p11.23). The *P* values [−log(*P*)] of all genotyped SNPs annotated with the gene structure are indicated. The best top SNP for ΔSBP120 is marked in purple and the rs code is also reported.
(PDF)

Figure S6 Graphic representation of SF2/ASF, SC35, SRp40 and SRp55 consensus motifs with respect to rs3790261 *A/G* polymorphism. Coloured bars represent high-score motifs of various binding factors. The height of each bar indicates the score value, the position along the x axis indicates its location along the sequence (*SLC24A3* intron3/exon4) and the width of the bar represents the length of the motif. The rs3790261 *A/G* at position +20 in exon 4 of *SLC24A3* and the relative SRp40 legend are boxed.
(PDF)

Figure S7 Regional plot of the *SLC8A1* and flanking region (chromosome 2 p22.1). The *P* values [−log(*P*)] of all genotyped SNPs annotated with the gene structure are indicated. The top ranking SNP for ΔSBP120 is marked in purple and the rs code is also reported.
(PDF)

Table S1 Genotype quality control data for Illumina 318K data.
(XLS)

Table S2 Association of SNPs in PRKG1, SLC24A3 and SLC8A1 with DDBP120, DSBP120 in stage 2 (n = 159).
(XLS)
