# Supplementary figures and images for: Genes Involved in Vasoconstriction and Vasodilation System Affect Salt-Sensitive Hypertension
Source: PLoS One. 2011 May 9;6(5):e19620. doi: 10.1371/journal.pone.0019620 (PMC3090407; doi:10.1371/journal.pone.0019620)

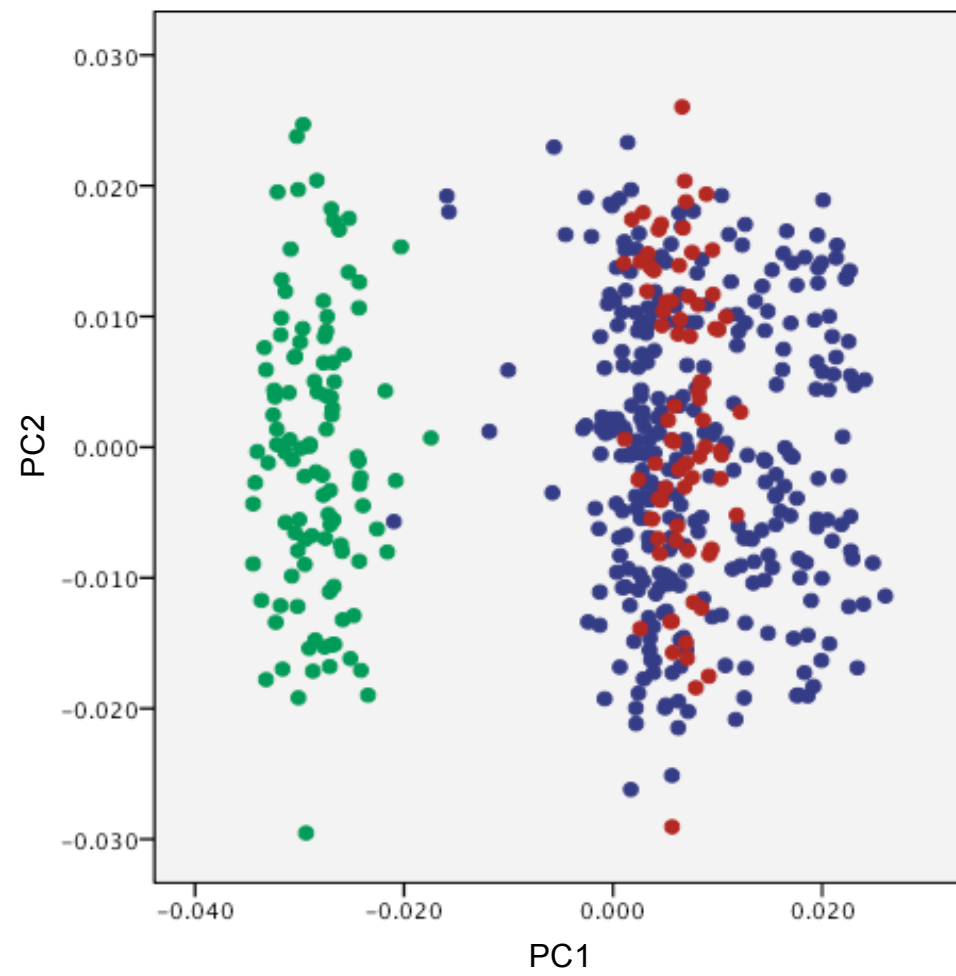

Supplement: Figure S1 — Multidimensional scaling plot of stage 1 cohort (blue circles), Utah residents with Northern and Western European ancestry population from the CEPH collection (green circles) and Tuscans (red circles) of HapMap data Release 27. The two principal components are plotted on the axis. (PDF) [file pone.0019620.s001.pdf]

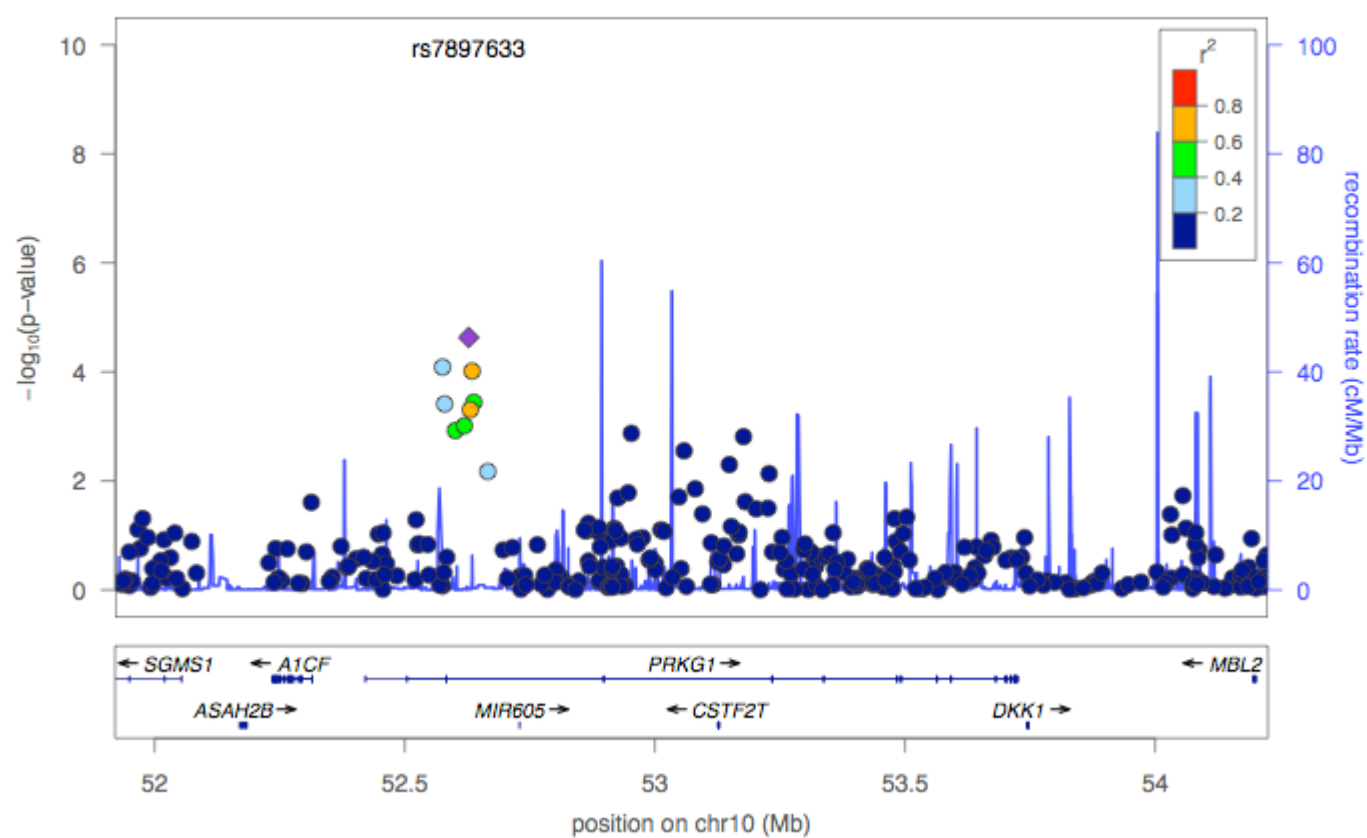

Supplement: Figure S2 — Regional plot of the PRKG1 and flanking region (chromosome 10 q11.23). The P values [−log(P)] of all genotyped SNPs annotated with the gene structure are indicated. The best top SNP for ΔDBP120 is marked with purple diamond and the rs code is also reported. The graph was drawn with LocusZoom software. (PDF) [file pone.0019620.s002.pdf]

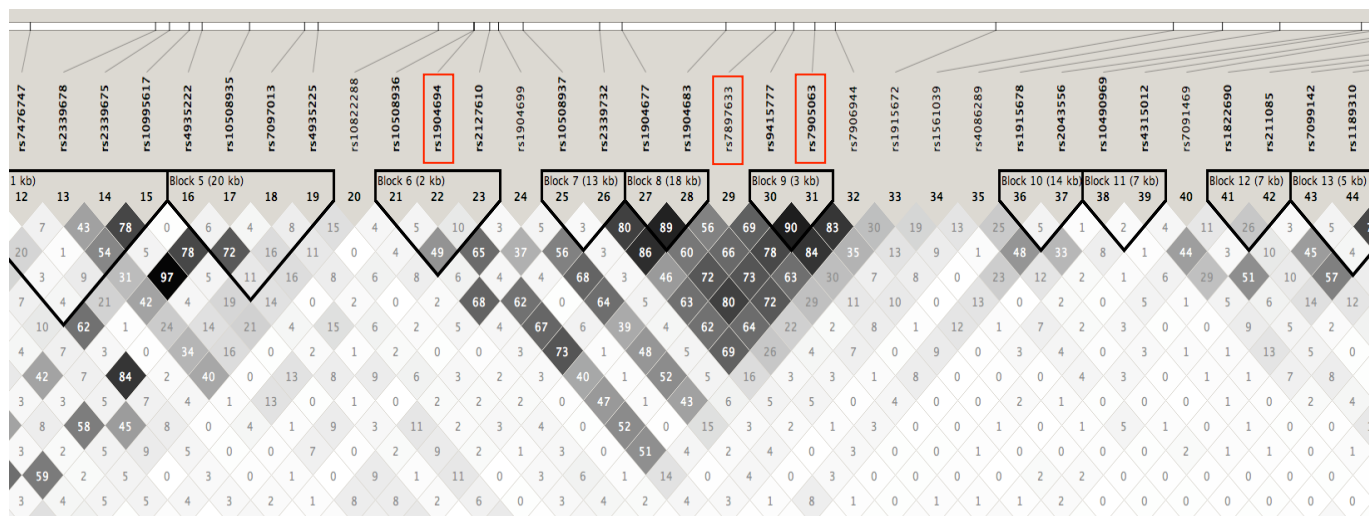

Supplement: Figure S3 — Map of the initial part of PRKG1 gene. The three independent SNPs are in the red box. The LD value (r2) between a specific pair of SNPs is shown within a corresponding “square”. The graph was drawn with Haploview software (http://www.broad.mit.edu/mpg/haploview). (PDF) [file pone.0019620.s003.pdf]

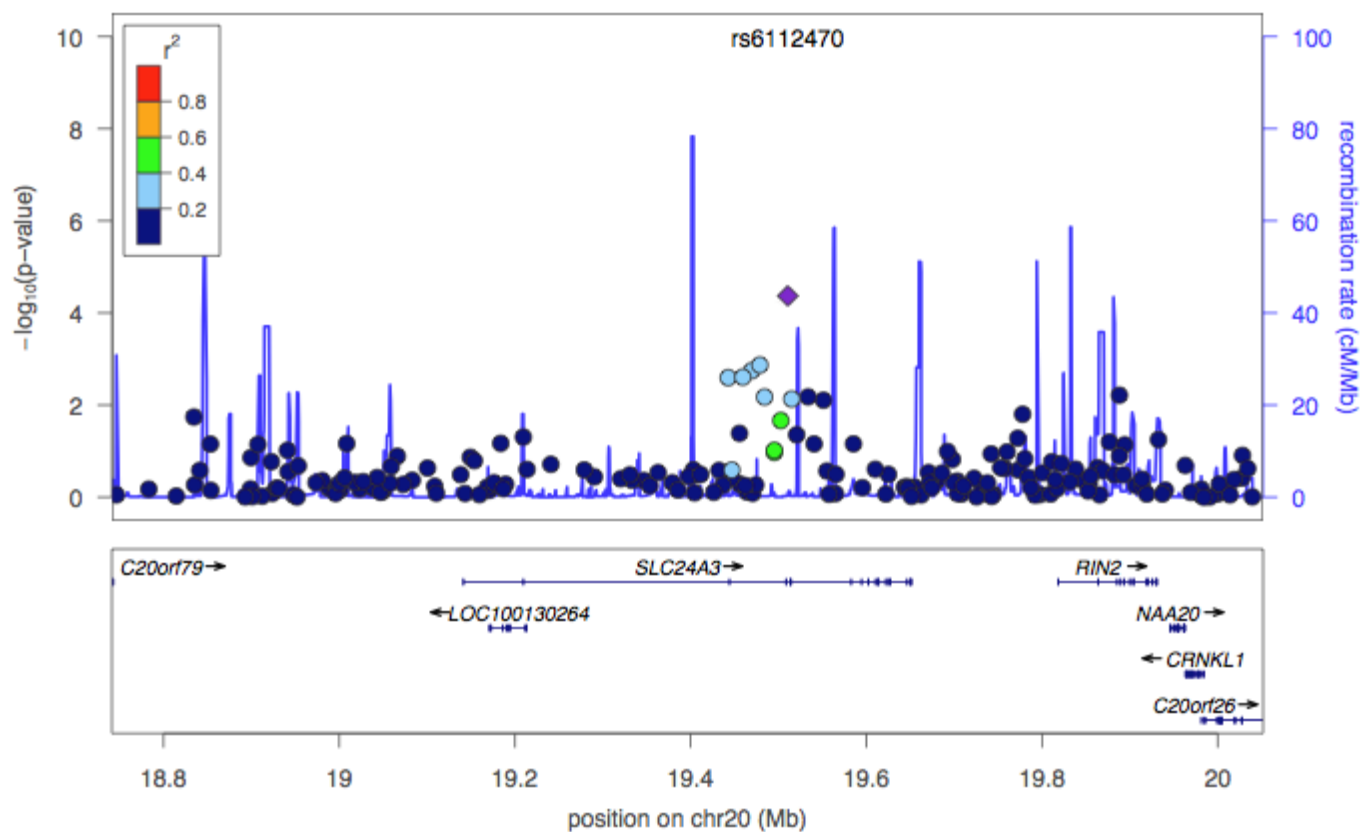

Supplement: Figure S5 — Regional plot of the SLC24A3 and flanking region (chromosome 20 p11.23). The P values [−log(P)] of all genotyped SNPs annotated with the gene structure are indicated. The best top SNP for ΔSBP120 is marked in purple and the rs code is also reported. (PDF) [file pone.0019620.s005.pdf]

# SRp40 binding

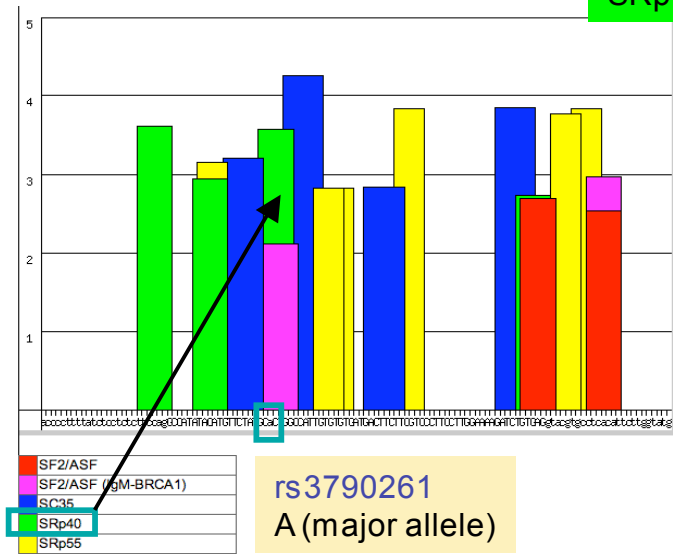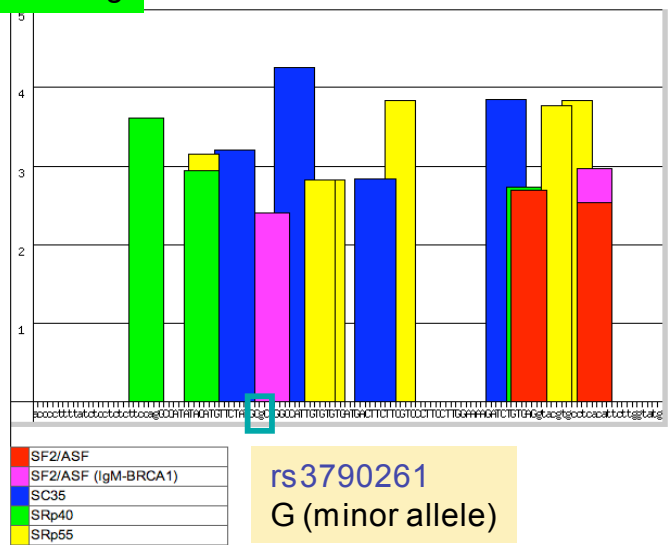

Supplement: Figure S6 — Graphic representation of SF2/ASF, SC35, SRp40 and SRp55 consensus motifs with respect to rs3790261 A/G polymorphism. Coloured bars represent high-score motifs of various binding factors. The height of each bar indicates the score value, the position along the x axis indicates its location along the sequence (SLC24A3 intron3/exon4) and the width of the bar represents the length of the motif. The rs3790261 A/G at position +20 in exon 4 of SLC24A3 and the relative SRp40 legend are boxed. (PDF) [file pone.0019620.s006.pdf]

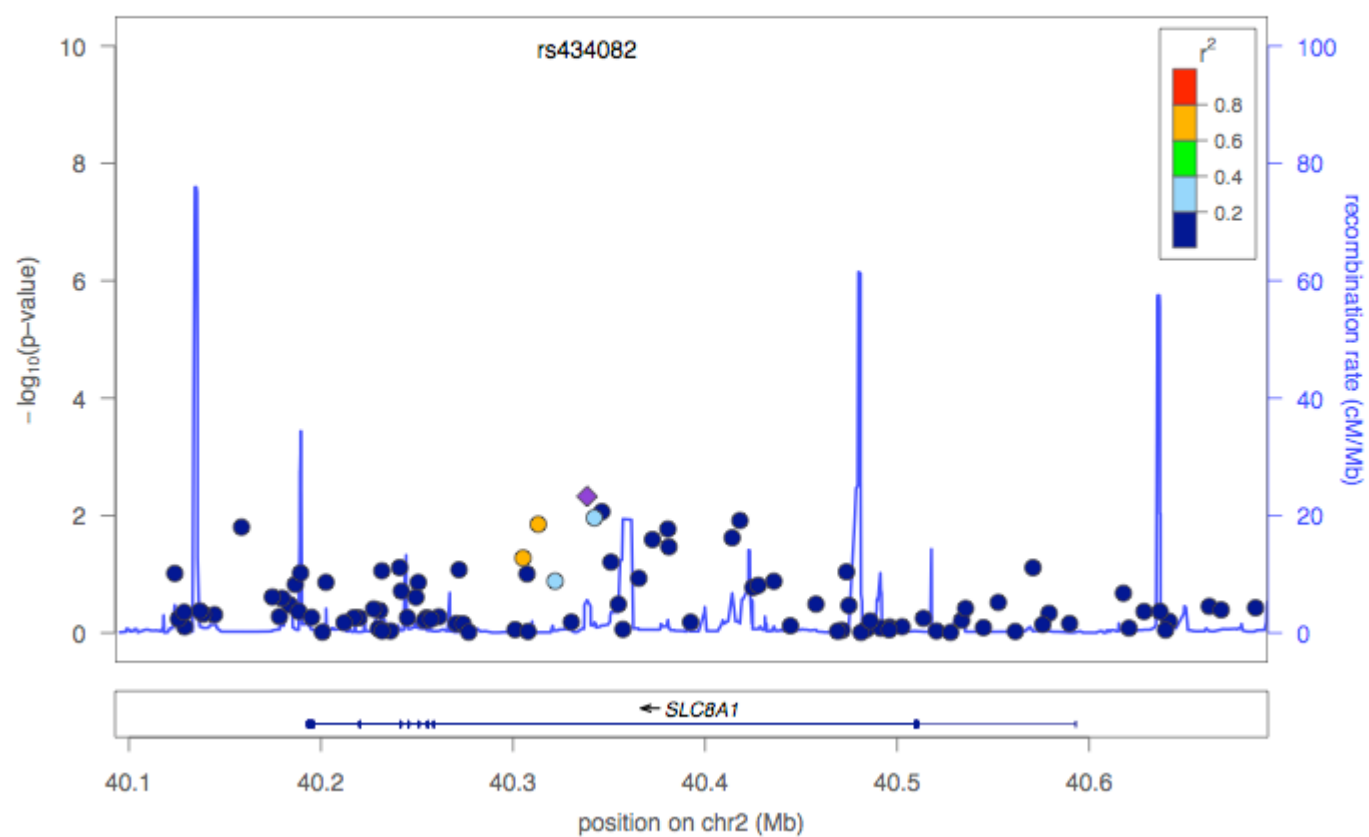

Supplement: Figure S7 — Regional plot of the SLC8A1 and flanking region (chromosome 2 p22.1). The P values [−log(P)] of all genotyped SNPs annotated with the gene structure are indicated. The top ranking SNP for ΔSBP120 is marked in purple and the rs code is also reported. (PDF) [file pone.0019620.s007.pdf]
