# Supplementary material for: Genes Involved in Vasoconstriction and Vasodilation System Affect Salt-Sensitive Hypertension
Source: PLoS One. 2011 May 9;6(5):e19620. doi: 10.1371/journal.pone.0019620 (PMC3090407; doi:10.1371/journal.pone.0019620)
Supplement: Figure S4 — Multiple sequence alignment of rs7897633 region in intron 2 of PRKG1. The highlighted area indicates SNP position. M means A/C transversion. Sequence alignment is provided by the UCSC Genome Browser (http://genome.ucsc.edu). (PDF) [file pone.0019620.s004.pdf]

52627700 - 52627757, 58 bps

```
  Human tttaaaagtgtttccataaagcccaa-----gMtgctttaaggaatcctctcaaatctgtggt
  Chimp tttaaaagtgtttccataaagcccaa-----gatgctttaaggaatcctctcaaatctgtggt
 Rhesus tttaaaagtgtttccataaagcccaa-----gatactttaaggaatcctctcaaatctgtggt
  Mouse tgtgagccagtttccatgatgccatagccatgatgcttcaaagattcttc-tgcaactgttct
    Rat tttaagccag-ttccatggtgccatagccatgatacttcaaggattcttc-tggaattgttgt
    Dog ============tctccgaagcccgc-----aacactccacggagc----------ctccttg
  Horse tttgagcttgtttccatgaagtccaa-----gatgctccgtggagtcttctcaaagctgttgt
    Cow tttaagttggtttccatgaagcccag-----gaagcttcctagaatcttctcaaagctgttgt
Opossum ===============================================================
```
